# Multivariate Matching Pursuit Decomposition and Normalized Gabor Entropy for Quantification of Preictal Trends in Epilepsy

**DOI:** 10.3390/e20060419

**Published:** 2018-05-31

**Authors:** Rui Liu, Bharat Karumuri, Joshua Adkinson, Timothy Noah Hutson, Ioannis Vlachos, Leon Iasemidis

**Affiliations:** 1 Mathematics and Statistics Department, Louisiana Tech University, Ruston, LA 71272, USA; 2 Biomedical Engineering Department, Louisiana Tech University, Ruston, LA 71272, USA

**Keywords:** Multivariate Matching Pursuit, Gabor entropy, complexity, Lorenz system, epilepsy

## Abstract

Quantification of the complexity of signals recorded concurrently from multivariate systems, such as the brain, plays an important role in the study and characterization of their state and state transitions. Multivariate analysis of the electroencephalographic signals (EEG) over time is conceptually most promising in unveiling the global dynamics of dynamical brain disorders such as epilepsy. We employed a novel methodology to study the global complexity of the epileptic brain en route to seizures. The developed measures of complexity were based on Multivariate Matching Pursuit (MMP) decomposition of signals in terms of time–frequency Gabor functions (atoms) and Shannon entropy. The measures were first validated on simulation data (Lorenz system) and then applied to EEGs from preictal (before seizure onsets) periods, recorded by intracranial electrodes from eight patients with temporal lobe epilepsy and a total of 42 seizures, in search of global trends of complexity before seizures onset. Out of five Gabor measures of complexity we tested, we found that our newly defined measure, the normalized Gabor entropy (NGE), was able to detect statistically significant (*p <* 0.05) nonlinear trends of the mean global complexity across all patients over 1 h periods prior to seizures’ onset. These trends pointed to a slow decrease of the epileptic brain’s global complexity over time accompanied by an increase of the variance of complexity closer to seizure onsets. These results show that the global complexity of the epileptic brain decreases at least 1 h prior to seizures and imply that the employed methodology and measures could be useful in identifying different brain states, monitoring of seizure susceptibility over time, and potentially in seizure prediction.

## 1. Introduction

Quantifying the complexity of physiological systems from their observable output signals is a multifaceted problem. Physiological systems are generally nonlinear, multiple input/output and massively coupled [1]. While the “complexity” of a system is oftentimes associated with the “variability” of its output signals, systems that exhibit high variability do not always exhibit high complexity [2]. Quantitative understanding of complexity is primarily accomplished using methods from information theory and statistics, with complexity being loosely associated with the amount of information needed to explain the system’s behavior [3]. Shannon entropy [4], approximate entropy [5], fractal dimension [6] and self-similarity [7,8,9] measures have been widely applied to the study of complexity of systems ranging from neuroscience to economics to ecology. In the majority of these applications, estimation of complexity is performed on the observable individual outputs (univariate signals). However, for systems with multiple outputs, this could lead to ambiguousness, as the univariate complexity measures of different outputs could produce conflicting results about the system’s complexity. For example, it has been argued in the literature that healthy biological systems are operating at high complexity and that aging and disease lead to decrease in their complexity [10]. This simplistic approach has been countered by investigations in changes in complexity associated with dynamics of diseases [11,12]. For example, in Alzheimer’s, brain complexity decreases [13], while schizophrenia and depression are associated with increase of brain’s complexity [14]. Based on the idea of Matching Pursuit decomposition of signals and concepts from information theory, we propose a methodology for estimation of the global complexity of multivariate systems, we show its potential to chaotic multivariate (low-dimensional but highly coupled) systems, and then apply it to the study of brain’s complexity during the transition into epileptic seizures.

The concept of Matching Pursuit (MP) was originally proposed by Mallat to extract patterns from noisy signals [15]. It is a greedy iterative process that decomposes a signal into individual components (atoms) based on an informative dictionary, which theoretically contains all possible basis functions (candidate atoms). The algorithm determines the best “matching” atoms, that is, the ones that contribute most to the creation of the original signal. MP is theoretically able to analyze intricate signals, such as continuous electroencephalogram (EEG), even when their dynamics are rapidly changing [16]. MP decomposition has been proven to be a useful tool for EEG time–frequency analysis including event-related desynchronization and synchronization (ERD/ERS) studies [17], detection of epileptic spikes [18] and seizure detection [19,20]. Analysis of EEG by MP using Gabor atoms as basis functions has led to Gabor Atom Density (GAD) as a measure of EEG’s complexity. This measure, together with multiple other measures estimating complexity, has been applied to detection of the transition to seizures with variable success [21,22]. MP, combined with other techniques, has also found application outside the field of epilepsy, for example in sleep spindles detection [23] and audio signal analysis [24].

The original MP algorithm was devised for univariate signal analysis and is thus capable of capturing a single signal’s information distributed across its own basis functions, and is not capable of capturing information distributed across basis functions determined jointly from many signals, for example, the outputs from a multivariate system. The Multivariate Matching Pursuit (MMP) method, which was more recently introduced [25] and later extended [26,27], can deal with this problem as it allows multi-dimensional (space–time–frequency) atomic decomposition [28,29]. With the addition of the extra dimension of space, MMP was shown to help with grouping similar activities across brain signals and quantifying differences in brain activation processes [30].

Epilepsy is a brain disorder characterized predominantly by recurrent and unprovoked interruptions of normal brain function (seizures). An epileptic seizure is a transient occurrence of abnormally excessive or synchronous neuronal activity in the brain [31]. Due to the unpredictable occurrence of epileptic seizures, patients with epilepsy are at high risk of injury in their daily life. It has been hypothesized that prior to seizures the dynamics of critical brain sites converge and diverge thereafter [32]. It was shown that seizures are preceded by abnormal, long-term and persistent synchronization of dynamics at those critical brain sites [32,33,34,35,36,37,38]. Spatial synchronization reduces the degrees of freedom of a system and may lead to decrease of its complexity. Nonetheless, the relation of synchronization and complexity is not straightforward [39,40]. Decrease of complexity has been reported prior to seizure occurrence in the epileptogenic focal area [41,42], employing a univariate analysis, that is, analysis per electrode site, and seizure. Employment of “global complexity” measures from multivariate analysis of EEG could offer new insights into the progression of the epileptic brain to seizures. Such an analysis has the potential to help us understand better the mechanisms of seizure generation (ictogenesis) and could find applications in the field of seizure prediction.

In this study, we developed measures of complexity based on Multivariate Matching Pursuit (MMP) decomposition of signals in terms of time–frequency Gabor functions (atoms) and Shannon entropy (Section 2). The measures were first validated on simulation data (Lorenz system) (Section 3.1). We then applied them to the EEGs from preictal (before seizure onsets) periods (Section 3.2) and investigated if there is any statistically significant short or long trend of complexity change (Hypothesis I and II respectively) of the brain en route to seizures common across seizures and patients. Discussion and conclusions of the obtained results are presented in Section 4 and Section 5, respectively.

## 2. Materials and Methods

### 2.1. Univariate Matching Pursuit Decomposition (MP)

MP is an iterative procedure that decomposes a signal into basis waveforms of sinusoids modulated by Gaussian envelopes (Gabor atoms) [10]. These basis functions constitute the dictionary of MP and have three parameters: scale (*s*), modulation frequency (*ξ*) and translation (*u*). The general form of a Gabor atom is given by:

(1) $$g_{s,\xi ,u\left(t\right)}=\frac{\sqrt[{4}]{2}}{\sqrt{s}}e^{-\pi {\left(\frac{t-u}{s}\right)}^{2}}e^{i\xi t}$$

In short, starting from the original univariate signal f(t), we find the basis function $g_{\gamma 0}\left(t\right),$ where $\gamma_{0}\;=\;\left(s_{0},\;\xi_{0},\;u_{0}\right)$ is selected from the basis function candidates in the dictionary, whose inner product with f(t) is maximum. Then, a residual signal $R^{1}f\left(t\right)$ is estimated from this fit of *f*(*t*) and the same procedure is repeated on the residual signals themselves. Therefore, in the (*n* + 1)^th^ level iteration, the MP algorithm selects the most suitable basis function $g_{\gamma n}\left(t\right)$ for the previous residual signal $R^{n}f\left(t\right)$, leaving a new residual signal $R^{n+1}f\left(t\right)$:

(2) $$R^{n+1}f\left(t\right)=R^{n}f\left(t\right)-C_{n}g_{\gamma n}\left(t\right),$$

where $\;\gamma_{n}\;=\;\left(s_{n},\;\xi_{n},\;u_{n}\right)$ and the Gabor coefficient *C_n_* equals the inner product $R^{n}f\left(t\right),g_{\gamma n}\left(t\right)$.

Thus, a univariate signal f(t) is decomposed into (*N* + 1) Gabor atoms $g_{\gamma n}\left(t\right)$, (n = 0, 1, 2,…,N), with their corresponding Gabor coefficients *C_n_* and the final residual:

(3) $$f\left(t\right)=\overset{N}{\underset{n=0}{\sum }}R^{n}f\left(t\right),g_{\gamma n}\left(t\right)g_{\gamma n}\left(t\right)+R^{N+1}f\left(t\right)\;=\overset{N}{\underset{n=0}{\sum }}C_{n}g_{\gamma n}\left(t\right)+R^{N+1}f\left(t\right)$$

Theoretically, for N→∞ and a complete dictionary, $\left|R^{N+1}f\left(t\right)\right|\to 0$ and MP leads to a perfect decomposition by selecting the best fit atoms $g_{\gamma n}\left(t\right)$. In practice, a stopping criterion for the number of MMP iterations is used by setting a threshold either on the number of iterations *N* (e.g., *N =* 200), or, on the ratio of the energy from the residual $\left|R^{N+1}f\left(t\right)\right|$ to the original signal |f(t)|.

### 2.2. Multivariate Matching Pursuit Decomposition (MMP)

The multivariate extension of MP, the Multivariate Matching Pursuit (MMP), uses the Gabor functions $g_{\gamma n}\left(t\right)$ from the dictionary to decompose an M-dimensional signal f(t,m), with m=1, 2, …,M, into atoms with Gabor coefficients $C_{n,m}$ such that:

(4) $$f\left(t,m\right)=\overset{N}{\underset{n=0}{\sum }}R^{n}f\left(t,m\right),g_{\gamma n}\left(t\right)g_{\gamma n}\left(t\right)+R^{N+1}f\left(t,m\right)\;=\overset{N}{\underset{n=0}{\sum }}C_{n,m}g_{\gamma n}\left(t\right)+R^{N+1}f\left(t,m\right)$$

Same stopping criteria for this iterative procedure can be applied to MMP as in MP [22]. In our analysis of the multivariate EEG, we used a 5% fixed energy ratio stopping criterion [43,44] with a fail-safe of a maximum of 200 iterations. With this criterion, we observed no decomposition of an EEG signal resulting into more than 200 atoms. Furthermore, a limited analysis using a 1% stopping criterion did not significantly change the results of the MMP decomposition. That is, the additional atoms had very small contribution to the total energy and negligible effect to the distribution of this energy.

### 2.3. MMP-Based Gabor Measures of Complexity

Based on MMP decomposition of multivariate data, five measures, the Gabor Atom Density (*GAD*) [13], the Gabor Mean Frequency (*GMF*), Gabor Energy (*GEn*), Gabor Entropy (*GE*), and the normalized Gabor Entropy (*NGE*) we have introduced [45] were estimated.

***GAD*** is the number of Gabor atoms selected by MMP from the basis functions dictionary in the decomposition of the original signals under the predetermined threshold criteria. *GAD* appears to be a “natural” measure of complexity under the assumption that more complex activity corresponds to higher number of atoms in the decomposed EEG signal. However, *GAD* treats all atoms equally and does not account for any difference in their characteristics, e.g., in their energy. ***GMF*** is the mean frequency that the Gabor atoms exhibit, estimated by averaging the modulation frequencies *ξ_i_* of each Gabor atom *i* in the decomposition.

Since the energy of a Gabor atom $g_{\gamma n}\left(t\right)$ in a decomposition of an *M*-dimensional signal is $\overset{M}{\underset{m=1}{\sum }}{\left|C_{n,m}\right|}^{2}$, and the total energy of all Gabor atoms (energy of the *M*-dimensional signal) ***GE_n_*** is

(5) $$GE_{n}=\overset{N}{\underset{n=0}{\sum }}\overset{M}{\underset{m=1}{\sum }}{\left|C_{n,m}\right|}^{2},$$

the relative energy of a Gabor atom is given by the ratio

(6) $$P_{n}=\frac{\sum_{m=1}^{M}{\left|C_{n,m}\right|}^{2}}{\sum_{n=0}^{N}\sum_{m=1}^{M}{\left|C_{n,m}\right|}^{2}},$$

where $P_{n}$ values are between 0 and 1.

$P_{n}$ can be considered as an energy distribution in the domain of Gabor atoms, and, via the Shannon entropy formulation, Gabor entropy (***GE***) can be defined as:

(7) $$GE=-\overset{N}{\underset{n=0}{\sum }}P_{n}\log_{2}P_{n}$$

This definition is similar to the one of the well-known spectral and wavelet entropies [46,47] but, in contrast to spectral and wavelet entropy, the thus defined *GE* depends on both the energy distribution $P_{n}$ and the number of atoms *N* + 1. The upper bound for *GE* in the extreme case, where $P_{n}$ are uniform $\left(P_{n}=\frac{1}{N+1}\right)$, is equal to ${log}_{2}\left(N+1\right)$. Based on this, we define the Normalized Gabor Entropy (***NGE***) as

(8) $$NGE=-\frac{\sum_{n=0}^{N}P_{n}\log_{2}P_{n}}{\log_{2}\left(N+1\right)}$$

Thus, *NGE* is a measure of the deviation of the energy distribution of Gabor atoms from uniformity, taking a value of 1 if $P_{n}$ are uniform. *NGE* is thus influenced only by the shape of the energy distribution among the atoms and not by the number of atoms (N + 1).

## 3. Results

The above measures estimated in the Gabor domain were first tested on simulation data generated from the classic three-dimensional Lorenz system in its chaotic regime [48]. We then applied them to multi-channel continuous iEEG data from patients with focal epilepsy, over the preictal period of a total of 42 recorded seizures across eight patients.

### 3.1. Simulation Data and Gabor Measures of Complexity

The well-known three-dimensional nonlinear Lorenz system in Equation (9), which models forced dissipative hydro dynamic flow and its chaoticity and complexity have been analytically studied, was employed to test the MMP algorithm and our Gabor measures of complexity. The differential equations of the Lorenz system are:

(9) $$\dot{x}=\sigma \left(y-x\right)\;\dot{y}=x\left(\rho -z\right)-y\;\dot{z}=xy-\beta z$$

**Figure 1:**
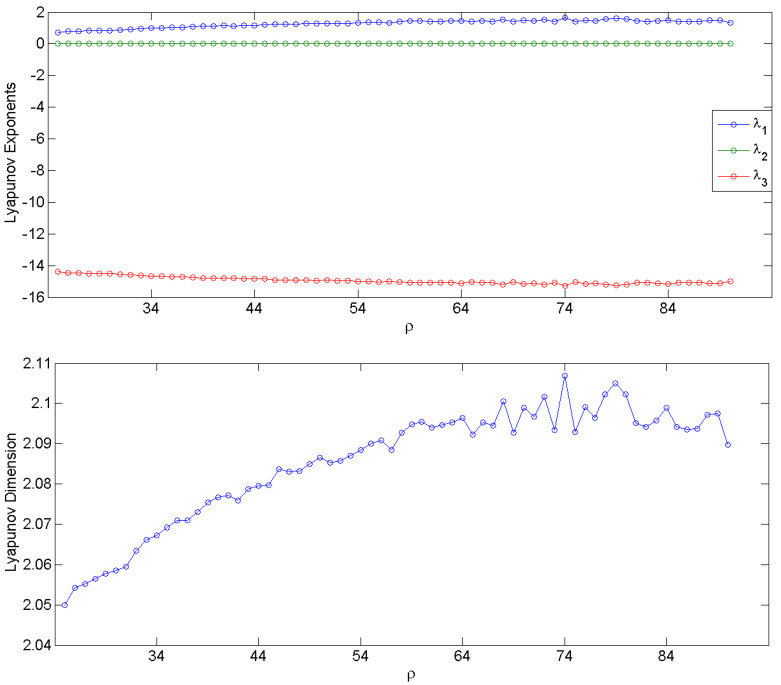
The three Lyapunov exponents of the 3-D Lorenz system (**top panel**) and the Lyapunov dimension (**bottom panel**) as a function of the model’s Rayleigh number  ρ.

Depending on the value of parameter ρ (Rayleigh number), the system can exhibit chaotic behavior of varying complexity. The Lyapunov exponents (measures of sensitivity to initial conditions and system’s stability) and the Lyapunov dimension (measure of complexity) were estimated numerically from the system of Lorenz ordinary differential equations (ODEs) [49,50] for integer values of ρ from 25 to 90 [51,52]. For ρ below 24 (not shown in Figure 1), the maximum Lyapunov exponent $\lambda_{1}$ is negative and the trajectories of the system in its state space converge to a fixed point; for integer ρ values between 25 and 90, $\lambda_{1}$ is positive, indicating chaotic behaviors, while the second largest Lyapunov exponent $\lambda_{2}$ is zero. The Lyapunov dimension is defined as $D_{L}=K+\frac{\sum_{\alpha =1}^{K}\lambda_{\alpha }}{\left|\lambda_{k+1}\right|}$, where *K* is the maximum number of Lyapunov exponents such that $\overset{K}{\underset{\alpha =1}{\sum }}\lambda_{\alpha }>0$ [53]. In the range 25 < ρ < 90, the Lorenz system has one positive Lyapunov exponent ($\lambda_{1}$), one equal to 0 ($\lambda_{2}$) and one negative ($\lambda_{3}$), and its Lyapunov dimension is $D_{L}=2+\frac{\lambda_{1}}{\left|\lambda_{3}\right|}$, which increases with ρ, reaching a relative plateau beyond ρ = 60. Both the Lyapunov exponents and the Lyapunov dimension for each value of ρ are shown in Figure 1.

**Figure 2:**
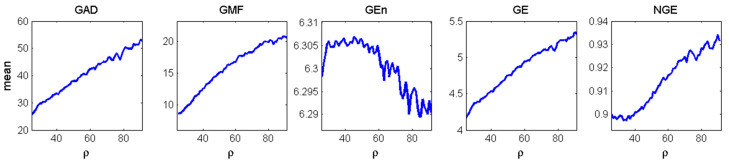
The mean values of the five MMP measures of complexity employed to characterize the evolution of the 3-D Lorenz system as a function of ρ (logarithmic scale is used for GEn values).

Next, we performed MMP analysis on the three-dimensional time series generated from the Lorenz system for every value of ρ and all five measures of complexity were estimated per time series. One thousand time series for (*x*, *y*, *z*) of 200 data points each were generated with 1000 randomly selected initial conditions for each ρ. The mean values of the measures over the 1000 simulations per ρ value are shown in Figure 2. They were then compared to the ones of the Lyapunov dimension in Figure 1 in search of common behavior as the complexity of the system changes with the change of ρ. Since each complexity measure has different units and range, e.g., the Lyapunov dimension is unitless and less than 3, *GAD* is measured in number of Gabor atoms (from 1 to 200), NGE is normalized from 0 to 1, etc., the trends in complexity with the increase of ρ were compared. As ρ increases, *GAD*, *GE*, *NGE* show the increase of the complexity of the system, in agreement with the complexity determined by the Lyapunov dimension. *GMF* also increases monotonically indicating that the increase in complexity of this system is accompanied with an increase in the central frequency of the signals. GEn remains constant up to ρ = 60, and decreases thereafter indicating that changes in the system’s complexity are not captured by measures such as GEn which accounts only for the variability (energy) of the signals.

### 3.2. Intracranial EEG Data

Continuous long-term intracranial EEG (iEEG) monitoring is regarded as the most valuable physiological data for studies of the epileptic brain [54]. The datasets analyzed in this study were multi-channel iEEG data from 1-h pre-seizure (preictal) periods of 42 clinical seizures recorded from eight patients with temporal lobe epilepsy (TLE) who underwent stereotactic placement of 40 or 28 bilateral depth and subdural electrodes. Preictal periods for analysis were selected only from all “isolated” clinical seizures in those patients, that is, clinical seizures that were at least 2-h apart. The available number of such seizures per patient varied between 2 and 14 with a median of 3.5 seizures per patient (see Table 1).

**Figure 3:**
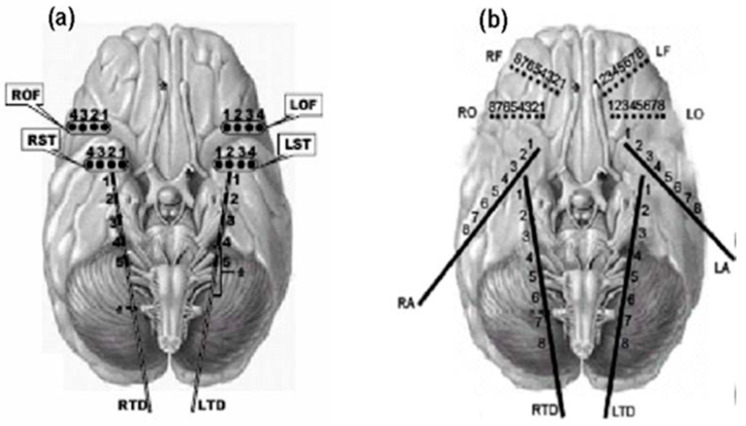
Electrode montages for the analyzed intracranial EEG recordings: (**a**) strip electrodes placed on the right and left orbitofrontal (ROF and LOF, respectively), and right and left subtemporal cortex (RST and LST, respectively) and depth electrodes on the right and left hippocampus (RTD and LTD, respectively); and (**b**) electrodes placed in same places as in (**a**) and additional depth electrodes placed on the right and left amygdala (RA and LA, respectively), and right and left frontal areas (RO and LO, respectively).

In seven out of the eight patients (Patients 2–8), 28 electrodes were placed bilaterally in the hippocampus, over the frontal and temporal lobes (Figure 3a). EEG signals were recorded with a Nicolet BMSI 4000 EEG machine using an average common reference and analog filtered with band-pass filter settings from 0.5 to 70 Hz. The data were subsequently sampled at 200 Hz. One of the eight patients (Patient 1) underwent stereotactic placement of 40 electrodes (Figure 3b) bilaterally in amygdala, mid hippocampus, orbitofrontal areas, and in frontal cortex from superior sagittal region near supplementary motor area. For this patient, the data were sampled at 400 Hz, but downsampled to 200 Hz for consistency in the data analysis across patients. The data were recorded at the University of Florida Health Shands Hospital in Gainesville, Florida (Patients 2–8) and the Barrow Neurological Institute in Phoenix, Arizona (Patient 1).

### 3.3. Estimation and Trend Analysis of Gabor Measures of Complexity from the EEG data

**Figure 4:**
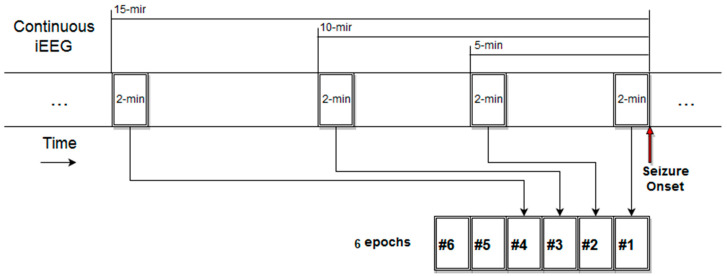
Diagrammatic representation of the temporal location of the six preictal EEG epochs that were analyzed from the available 1 h preictal iEEG recordings per seizure and patient.

Six preictal EEG epochs of 2 min in duration each were analyzed at 2, 5, 10, 15, 30 and 60 min before each seizure (see Figure 4 for placement of those epochs in the time axis) with the methodology described in Section 2 and with *M* = 28 or 40 variables (electrodes) depending on the number of implanted electrodes per patient. The five MMP-based measures were estimated from 1-s non-overlapping EEG segments for each of the six epochs (i.e., 120 measure values per epoch).

To identify any preictal trends in brain’s complexity from the analysis of the multivariate EEG, we employed model fitting procedures and subsequent statistical analysis to determine the statistical significance of any identified trends. If we denote by *i* the patients and $s_{i}\;\left(i\;=\;1,\ldots ,\;I\right)$ the number of seizures for patient *i*, the pair (i, j) with $j\;=\;1,\ldots ,\;s_{i}$ represents the *jth* seizure of the *ith* patient. For a given measure of complexity that takes values y(t) during a given time period *t* of 1-s duration, a trend coefficient $\alpha_{i,j}$ was estimated per seizure *j* and patient *i* by fitting one of three basic monotonic trend models to y(t) within the time period (one epoch or multiple epochs), we want to consider: two curve models $m1\;:\;y_{t}\;=\;C_{i,j}t^{\alpha_{i,j}}\;$ (power law) and $m2\;:\;y_{t}\;=\;C_{i,j}e^{\alpha_{i,j}}{}^{t}$ (exponential), and one linear model $m3\;:\;y_{t}\;=\;C_{i,j}\;+\;\alpha_{i,j}t$. Subsequently, for each measure separately, the optimal model type (*m*1, *m*2 or *m*3) was selected based on minimization of the mean squared fitting error across all seizures and patients.

The estimated trend coefficients $\alpha_{i,j}$ from the selected model were then averaged across seizures for each patient separately, $a_{i}=\frac{1}{s_{i}}\overset{s_{i}}{\underset{j=1}{\sum }}a_{i,j},$ to derive a characteristic preictal trend value $\alpha_{i}$ per patient *i*. Regardless of the model type, an $\alpha_{i}$ value close to 0 indicates no trend, while positive/negative values indicate increasing/decreasing trends, respectively. The intercept terms $C_{i,j}$ in the models can significantly vary from patient to patient or even from seizure to seizure in the same patient. They correspond to “baseline” values of a complexity measure and are independent of trends. Hence, by concentrating only on the trend coefficients $\alpha_{i}$ we do not consider the across patients or within patient variability of a complexity measure due to its values in the “interictal” (baseline) brain states. Finally, statistical tests were employed to test the significance of the $\alpha_{i}$ values. Lilliefors goodness-of-fit test was used to check the normality of $\alpha_{i}$’s and as a guide in the use of t-test or Sign test [55,56]. False Discovery Rate (FDR) adjustment of *p*-values with *q* = 0.05 was performed on the obtained statistical tests’ *p*-values [57,58] to control for type II error (i.e., false identification of significant trend) due to multiple comparisons.

Two hypotheses about existence of preictal trends were tested with the use of the above trend analysis of brain’s global complexity Gabor measures: **Hypothesis (I)**, existence of “short-term” trend during the one 2-min EEG epoch immediately preceding seizures onset; and **Hypothesis (II)**, existence of “long-term” trend along the six epochs over the 60-min period prior to seizures.

**Test of Hypothesis I:** The 120 values derived from each measure within the one 2-min epoch immediately prior to seizures’ onset were used in the trend analysis. The trend coefficients $\alpha_{i}$s that were estimated only within this epoch per patient were used in the modeling with the three models to quantify any characteristic preictal (short-term) trends per patient within this epoch, and then test the null hypothesis of no statistically significant average trend $\bar{\alpha }$ across patients in this epoch.

For Hypothesis (I), the investigation of short-term trends in the 2-min epoch immediately preceding a seizure did not result to identification of any statistically significant trends (see Table 2). After optimization, the selected models for trend analysis for all measures were found to be of the linear form (*m*3), and for each measure the average trend coefficients ($\bar{\alpha }$) over the 120 s and patients were not different from zero (FDR adjusted *p* > 0.05).

**Test of Hypothesis II:** We followed the following steps: (1) Estimate the mean *μ* and the standard deviation *σ* of the 120 values per epoch for each of the six pre-seizure epochs per seizure. (2) Apply the model fitting (*m*1, *m*2, or *m*3) on those mean and standard deviation values per seizure to derive the long-term preictal trends $a_{i,j}$ per seizure over time. (3) Average $a_{i,j}$ across seizures to derive the $\alpha_{i}$ for *μ* and *σ* per patient *I*. (4) Estimate the statistical significance of the average $\bar{\alpha }$ of $\alpha_{i}$ for both *μ* and *σ* across all patients as in testing Hypothesis (I). Under this framework, we sought to detect any long preictal trends (within 1 h prior to seizures) in the mean and standard deviation of the complexity values that were common across seizures and patients. In the test of this hypothesis we used an uneven sampling of epochs before a seizure, selecting more epochs closer than farther away from seizure onsets. The rationale for this non-uniform sampling is that EEG epochs closer to seizure onset are expected to contain more information relevant to transition to impending seizure than ones farther away from seizure onset. In addition, farther away from seizure onset epochs are expected to produce more spurious (i.e., irrelevant with respect to a trend in) values of complexity and, thus, the more such epochs we include in our trend analysis the more noisy and difficult to identify any trends would be. In addition, given that preictal periods cannot be accurately determined and may vary in duration per seizure in the same patient as well as across patients, uniform sampling of epochs over a long (e.g., 1 h) period prior to seizures may increase the probability of inclusion of interictal (normal) brain activity that would mask real preictal activity.

**Figure 5:**
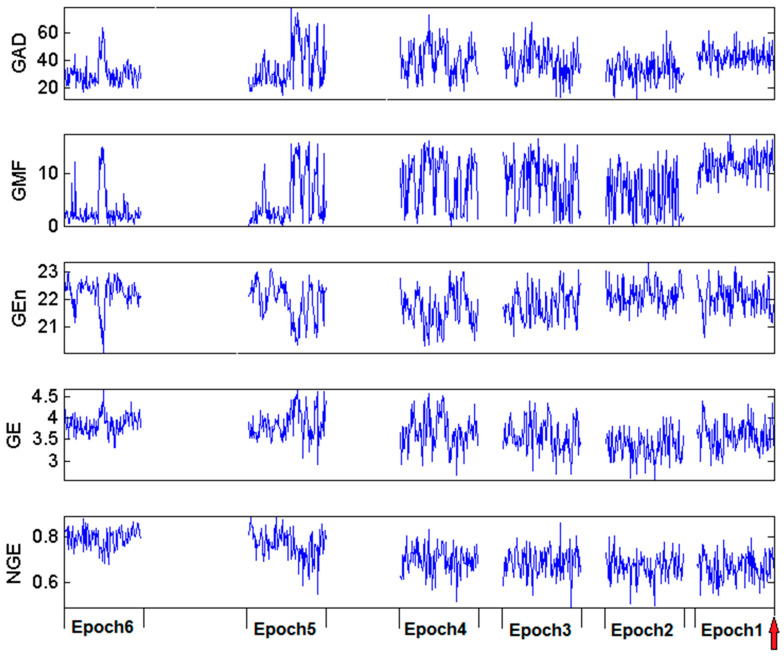
Complexity values per Gabor measure within each of six EEG epochs prior to Seizure 6 of Patient 2. Epochs are 2-min in duration and each measure value was estimated from 1-s non-overlapping EEG segments within each epoch (120 measure values per epoch).

The estimated 120 values of the Gabor complexity measures *GAD*, *GMF*, *GEn*, *GE*, *NGE* within each of the six EEG epochs before a clinical seizure in one of our patients are shown in Figure 5. Variations of the values (short term variability) are observed for almost all measures. By visual inspection of this figure, a lack of consistency of a trend across epochs is observed in all Gabor measures of complexity except for *NGE*. In particular, *GAD* shows high values in Epochs 5, 4, 3 and 1; *GMF* shows highest values in Epoch 1; *GEn* shows no visible differences across epochs; and *GE* shows a downward trend up to Epoch 1 at which it takes higher values. The normalized Gabor entropy (NGE) exhibits a consistent trend across epochs, that is, a slow but progressive drop across epochs of the (visually estimated) mean of *NGE* values per epoch as the seizure onset approaches.

For Hypothesis (II), the models that optimally fit the means *μ* and standard deviations *σ* per measure are given in Table 3. These results from the model fitting per measure in the 1-h preictal periods across seizures and patients indicate that mean and standard deviation of *GAD* and *GE* exhibit power law behavior over time, while *GEn* and *NGE* exhibit power law behavior for their means, and exponential behavior for their standard deviations. *GMF* exhibits linear behavior for both its mean and standard deviation over time.

In the first eight columns of Table 4, the long-term trends $\alpha_{i}$ for the mean (*μ*) and standard deviation (*σ*) per measure and patient are given. The corresponding $\bar{\alpha }$ for the long-term trends of the mean (*μ*) and standard deviation (*σ*) per measure across patients were then tested for statistical significance (i.e., test against being zero across patients) using one-sample t-tests or sign tests depending on the existence/absence of normality of their values respectively. Each of these tests was applied on sets of eight $\alpha_{i}$ values across patients (one $\alpha_{i}$ value per patient *i*) from which the statistical significance of the preictal trends of the mean and standard deviation of each measure were estimated (given in the last column of Table 4). In three cases (μ(*GAD*), μ(*GMF*) and σ(*GMF*)), normality was rejected by Lilliefors test and sign tests were used, while for the rest of the cases t-tests were employed. All standard deviations (σ(*GAD*), σ(*GMF*), σ(*GEn*), σ(*GE*), and σ(*NGE*)) and only one mean value (μ(*NGE*)) of complexity measures exhibited statistically significant preictal trends across patients at the significance level of 0.05.

## 4. Discussion

We developed a new measure, the normalized Gabor entropy (*NGE*), to quantify the global complexity of a multivariate system based on MMP and Shannon entropy. In simulation data from a well-known multivariate chaotic system (Lorenz system), we have shown that NGE is capable of capturing well the changes in the system’s complexity as a function of its parameters. Application of this approach to real EEG data from patients with epilepsy showed that a statistically significant (*p* < 0.05) nonlinearly decreasing trend in brain’s complexity develops over a period of 1 h before seizures’ occurrence (Hypothesis (II)) across seizures and patients. We found no statistically significant short-term trends (within 2-min prior to seizures’ onset—Hypothesis (I)) across seizures and patients. Taken together the above results indicate that preictal changes happen either at a very slow rate or much earlier than 2 min prior to seizure onset.

All Gabor measures of complexity tested showed statistically significant (*p <* 0.05) long-term increase in their standard deviation. This could potentially be problematic when trying to accurately identify preictal changes based on the measure values themselves. On the other hand, this increase in variation of complexity might be used as an indication of an impending seizure.

Some Gabor measures showed gradual decreasing or increasing trends for certain seizures (e.g., *GE* for Seizure 6 of Patient 2 shown in Figure 5), but these phenomena did not survive statistical testing across seizures and patients. The measure of Gabor atom density (*GAD*), that has shown promise in the literature based on single channel, did not perform satisfactory in identifying statistically significant preictal trends in this multivariate approach.

With respect to uneven sampling we used in this study, if the analysis were prospective (e.g., seizure prediction analysis), there would be no other choice than using uniform sampling (no a priori knowledge of when the seizure would occur). Our methodology was applied to data from eight patients, and the selection of 1 h per seizure for analysis was based on previously reported average preictal period values in the literature [59,60,61,62]. Unfortunately, identification of the preictal period is still an open problem in the scientific community [63], and uneven sampling (a “weighted” approach) of preictal periods is one way to address this ambiguity and its potentially adverse effect on analysis.

Limitations of our analysis include the use of general enough but still of particular trend-type models (linear, exponential or power law) in the search of common preictal trends in brain’s complexity across seizures and patients. There is also the possibility that characteristic changes in complexity are patient or seizure-type (e.g., partial or generalized seizure) specific. Then, a more adaptive, patient–seizure–measure specific scheme may be able to better detect preictal trends of complexity. Finally, there is a possibility of using our developed NGE measure as a new feature in machine learning-based systems to improve the performance of seizure prediction algorithms [64].

## 5. Conclusions

We presented a novel methodology and measures to study the complexity of the epileptic brain based on the decomposition of observed EEG into distinct Gabor atoms and their entropy. This methodology can be employed in the study of multivariate systems in general, ranging from physiological to geophysical to economical systems. Application of our methodology to analysis of multichannel EEG signals recorded from patients with temporal lobe epilepsy during the transition to seizures showed a statistically significant (*p <* 0.05) progressive decrease of brain’s spatio-temporal complexity prior to seizures’ onset across seizures and patients. This finding, together with the methodology we developed, could be utilized to improve existing algorithms for timely prediction of seizure occurrences, monitor long-term trends of patients’ susceptibility to seizures, in better characterizing and identifying brain states of critical significance in other than epilepsy brain disorders (e.g., Parkinson’s and Alzheimer’s diseases) as well as in the normal function of the healthy brain (e.g., cognition states).

## Figures and Tables

**Table 1:** Patient information and available iEEG datasets.

Patient	Gender	# Recording Electrodes	Available iEEG Duration (hours)	Number of Isolated Clinical Seizures
1	F	40	34.67	4
2	M	28	281.68	6
3	F	28	86.3	14
4	M	28	334.62	7
5	M	28	85.02	3
6	M	28	156.22	2
7	M	28	145.77	3
8	F	28	18.77	3

**Table 2:** Short-term preictal trend $\bar{\alpha }$ across patients for Hypothesis (I).

Measure	Model	$\bar{\alpha }$	FDR Adjusted *p*-Value
GAD	*m*3	0.0092	1
GMF	*m*3	0.0032	1
GEn	*m*3	$8.2920\;\times \;10^{-5}$	1
GE	*m*3	$3.0526\;\times \;10^{-5}$	1
NGE	*m*3	$-2.7492\;\times \;10^{-5}$	1

**Table 3:** The optimally selected models (*m*1, *m*2 or *m*3) for identification of long-term (across epochs) trends $\alpha_{i}$ in μ and σ profiles per Gabor complexity measure.

Complexity Measure	*GAD*	*GMF*	*GEn*	*GE*	*NGE*
Statistic of Measure	Optimized Model for Trend Identification of Statistic across Epochs				
μ	*m*1	*m*3	*m*1	*m*1	*m*1
σ	*m*1	*m*3	*m*2	*m*1	*m*2

**Table 4:** Long-term preictal trends across patients for Hypothesis (II).

Patient	P1 (α_1_)	P2 (α_2_)	P3 (α_3_)	P4 (α_4_)	P5 (α_5_)	P6 (α_6_)	P7 (α_7_)	P8 (α_8_)	$\bar{\alpha }$	FDR Adjusted *p*-Value for $\bar{\alpha }$ Significance
Statistic (Measure)										
μ(*GAD*)	0.0242	−0.0102	0.479	−0.0173	−0.0097	−0.0213	−0.0215	0.1185	0.0138	0.7266
σ(*GAD*)	0.031	0.0241	0.0248	0.0424	0.0501	0.0467	0.0237	0.0588	0.0316	0.0206
μ(*GMF*)	0.0177	0.0089	0.0209	−0.0031	−0.0047	−0.0017	−0.0079	0.052	0.0103	0.2359
σ(*GMF*)	0.0076	0.0042	0.0025	0.003	0.0036	0.0013	0.0026	0.0243	0.0061	0.0195
μ(*GEn*)	−0.0009	0.0063	−0.0035	0.0088	0.0038	0.0023	0.0126	−0.0018	0.0034	0.1773
σ(*GEn*)	0.0013	0.0034	0.0014	0.002	0.0024	0.0005	0.0062	0.0055	0.0028	0.0195
μ(*GE*)	−0.0018	−0.0082	0.0141	−0.0147	−0.0035	−0.019	−0.0162	0.0252	−0.003	0.6645
σ(*GE*)	0.0471	0.0201	0.021	0.0777	0.0371	0.0652	0.06	0.1004	0.0536	0.0097
μ(*NGE*)	−0.0077	−0.0074	−0.001	−0.0091	0	−0.0117	−0.0098	−0.0176	−0.008	0.0195
σ(*NGE*)	0.0017	0.0014	−0.0004	0.0027	0.0024	0.0039	0.004	−0.001	0.0018	0.0409
